# Evidence of New Risk Genetic Factor to Systemic Lupus Erythematosus: The *UBASH3A* Gene

**DOI:** 10.1371/journal.pone.0060646

**Published:** 2013-04-02

**Authors:** Lina-Marcela Diaz-Gallo, Elena Sánchez, Norberto Ortego-Centeno, Jose Mario Sabio, Francisco J. García-Hernández, Enrique de Ramón, Miguel A. González-Gay, Torsten Witte, Hans-Joachim Anders, María F. González-Escribano, Javier Martin

**Affiliations:** 1 Cellular Biology and Immunology Department, Instituto de Parasitología y Biomedicina “López-Neyra”, Consejo Superior de Investigaciones Científicas (IPBLN- Consejo Superior de Investigaciones Científicas), Granada, Spain; 2 Department of Internal Medicine, Hospital Clínico San Cecilio, Granada, Spain; 3 Department of Internal Medicine, Hospital Virgen de las Nieves, Granada, Spain; 4 Department of Internal Medicine, Hospital Virgen del Rocío, Sevilla, Spain; 5 Department of Internal Medicine, Hospital Carlos Haya, Málaga, Spain; 6 Department of Rheumatology, Instituto de Formación e Investigación Marqués de Valdecilla, Hospital Universitario Marqués de Valdecilla, Santander, Spain; 7 Department of Clinical Immunology and Rheumatology, Hannover Medical School, Hannover, Germany; 8 Medical department and policlinic IV, Klinikum der Universität, München, Munich, Germany; 9 Department of Immunology, Hospital Virgen del Rocío, Sevilla, Spain; MOE Key Laboratory of Environment and Health, School of Public Health, Tongji Medical College, Huazhong University of Science and Technology, China

## Abstract

The ubiquitin associated and Src-homology 3 (SH3) domain containing A (*UBASH3a*) is a suppressor of T-cell receptor signaling, underscoring antigen presentation to T-cells as a critical shared mechanism of diseases pathogenesis. The aim of the present study was to determine whether the *UBASH3a* gene influence the susceptibility to systemic lupus erythematosus (SLE) in Caucasian populations. We evaluated five *UBASH3a* polymorphisms (rs2277798, rs2277800, rs9976767, rs13048049 and rs17114930), using TaqMan® allelic discrimination assays, in a discovery cohort that included 906 SLE patients and 1165 healthy controls from Spain. The SNPs that exhibit statistical significance difference were evaluated in a German replication cohort of 360 SLE patients and 379 healthy controls. The case-control analysis in the Spanish population showed a significant association between the rs9976767 and SLE (Pc = 9.9E-03 OR = 1.21 95%CI = 1.07–1.37) and a trend of association for the rs2277798 analysis (P = 0.09 OR = 0.9 95%CI = 0.79–1.02). The replication in a German cohort and the meta-analysis confirmed that the rs9976767 (Pc = 0.02; Pc = 2.4E-04, for German cohort and meta-analysis, respectively) and rs2277798 (Pc = 0.013; Pc = 4.7E-03, for German cohort and meta-analysis, respectively) *UBASH3a* variants are susceptibility factors for SLE. Finally, a conditional regression analysis suggested that the most likely genetic variation responsible for the association was the rs9976767 polymorphism. Our results suggest that *UBASH3a* gene plays a role in the susceptibility to SLE. Moreover, our study indicates that *UBASH3a* can be considered as a common genetic factor in autoimmune diseases.

## Introduction

The T cell ubiquitin ligand proteins (TULA) family is characterized by function as suppressors of T cell receptor signalling. One of the members of the TULA family proteins is the ubiquitin associated and Src-homology 3 (SH3) domain containing A (*UBASH3a*) which is expressed only in lymphoid cells and facilitates apoptosis induced in T cells by certain stimuli, such as growth factor withdrawal [1]. *UBASH3a* gene spans 40 kb, contains 15 exons and is located on human chromosome 21q22.3 [2]. The lack of TULA proteins resulted in hyper-reactivity of T cells [1]. Evidence for both B and T lymphocyte hyper-reactivity is typically observed in autoimmune disorders [2]. These disorders are characterized by an inappropriate, ultimately excessive, inflammatory response against self, resulting in tissue destruction. Although many individuals affected by autoimmune diseases demonstrate multiorgan involvement, the primary end-organ target (e.g., autoimmune destruction of pancreatic islet cells in type 1 diabetes mellitus) typically drives the clinical presentation and disease definition. Recent studies have showed that single nucleotide polymorphisms (SNPs) of the *UBASH3a* gene are associated with some autoimmune diseases, like type 1 diabetes (T1D), celiac disease (CD), rheumatoid arthritis (RA) and vitiligo, suggesting that this gene could play an important role in the pathogenesis of autoimmune disorders [3]–[8].

Systemic lupus erythematosus (SLE) is a prototypic autoimmune diseases characterized by the production of autoantibodies, immune-complex deposition, and subsequent multiple organ damage. The complex aetiology of autoimmune diseases includes environmental, hormonal and genetic factors. Some of those factors remained to be defined [3], [4]. Based on these insights, the aim of the present study was to evaluate the role of five *UBASH3a* polymorphism in SLE.

## Materials and Methods

### Ethics Statement

Written informed consent was obtained from all participants and the respectively ethics committee approved the study according to the principles expressed in the Declaration of Helsinki.

The case-control study included 906 SLE patients and 1165 healthy controls from a white Spanish population. The replication cohort from white Germans comprehends 360 SLE patients and 379 healthy controls. All the patients met the American College of Rheumatology criteria for classification of SLE [5]. Written informed consent was obtained from all participants and the respectively ethics committee approved the study. DNA was obtained from peripheral blood using standard methods. The samples were genotyped for the *UBASH3a* rs2277798, rs2277800, rs9976767, rs13048049 and rs17114930 polymorphisms via TaqMan® 5′allelic discrimination technology using a predesigned SNPs genotyping assays provided by Applied Biosystems (assay ID: C___1724055_10, C__15885522_20, C___1724067_10, C___1724073_20 and C___25622591_10, respectively; Figure S1). At the moment of the design of the study the only confirmed case-control associated SNP with autoimmune diseases was the rs9976767 [6]. The other four SNPs were selected because they were not included in previous SLE genetic studies and they are non-synonymous changes located in different exons of the *UBASH3a* gene. Moreover, the minor allele frequency (MAF) of those SNPs was reported in Caucasian populations and they exhibited moderated LD with at least one SNP in the loci. Deviation from Hardy-Weinberg equilibrium (HWE) was tested by standard chi-square analysis. The differences in genotype distribution and allele frequency among cases and controls were calculated by contingency tables and when necessary by Fisher's exact test. Odds ratios (OR), and 95% confidence intervals (CI), were calculated according to Woolf's method. Combined data were analysed by Mantel-Haenszel tests under fixed effect model and the Breslow-Day (BD) test was used to estimate the OR heterogeneity amongst the two cohorts. An association was considered statistically significant if P<0.05. Benjamini & Hochberg (1995) step-up false discovery rate (FDR) control correction [7] for multiple testing was applied to the P-values in both the independent analysis and the combined meta-analysis (P_c_). Linkage disequilibrium (LD) measurement (r^2^) between the studied SNPs was estimated by expectation-maximization algorithm using HAPLOVIEW (version 4.2; Broad Institute of MIT and Harvard). Finally, the dependency of the association between each SNP and every studied genetic variant was determined by a conditional logistic regression analysis (considering the different cohorts as covariate). The analyses were performed using PLINK (version 1.07) [8].

## Results

The distributions of genotypic and allelic frequencies of the five *UABSH3a* evaluated polymorphisms were in HWE at 5% significance level. Additionally, MAFs of the studied SNPs were similar to those reported by the HapMap project for the CEU population (http://hapmap.ncbi.nlm.nih.gov/) in both, Spanish and German cohorts. The LD structure of the five *UABSH3a* SNPs in the Spanish cohort is shown in (Figure S1). The Table 1 summarizes the results of the association analysis for the discovery cohort. The minor allele of the rs9976767 polymorphism exhibited a statistical significant association with SLE in the Spanish population (P_c_ = 9.9E-03, OR = 1.21, 95%CI = 1.07–1.37). In addition we observed a trend of association with the rs2277798 polymorphism (P = 0.099, P_c_ = 0.248, OR = 0.9, 95%CI = 0.79–1.02). The frequency of the minor alleles of the rs2277800, rs13048049 and rs17114930 *UBASH3a* polymorphisms were not statistically significantly different between SLE patients and healthy controls in the Spanish cohort.

**Table 1:** Genotype and minor allele frequencies of *UBASH3a* SNPs located in Caucasian SLE patients and healthy controls from Spain, the discovery cohort.

			Genotype, N (%)			Alleles, N(%)		Allele test	
**SNP**	**1/2**	**Subgroup (N)**	**1/1**	**1/2**	**2/2**	**1**	**2**	***P*** **-value** *	OR [CI 95%]****
rs2277798	G/A	Controls (n = 1165)	477 (40.94)	529 (45.41)	159 (13.65)	1483 (63.6)	847 (36.4)		
		SLE (n = 906)	402 (44.37)	394 (43.49)	110 (12.14)	1198 (66.1)	614 (33.9)	0.0993**	0.90 [0.79-1.02]
rs2277800	C/T	Controls (n = 1165)	1080 (92.70)	84 (7.21)	1 (0.09)	2244 (96.3)	86 (3.7)		
		SLE (n = 906)	832 (91.83)	73 (8.06)	1 (0.11)	1737 (95.9)	75 (4.1)	0.4592	1.13 [0.82–1.55]
rs9976767	A/G	Controls (n = 1165)	363 (31.16)	558 (47.90)	244 (20.94)	1284 (55.1)	1046 (44.9)		
		SLE (n = 906)	230 (25.39)	451 (49.78)	225 (24.83)	911 (503)	901 (49.7)	1.99E-03***	1.21 [1.07–1.37]
rs13048049	G/A	Controls (n = 1165)	1038 (89.10)	126 (10.82)	1 (0.09)	2202 (94.5)	128 (5.5)		
		SLE (n = 906)	808 (89.18)	96 (10.60)	2 (0.22)	1712 (94.5)	100 (5.5)	0.9719	1.01 [0.77–1.32]
rs17114930	C/G	Controls (n = 1165)	1066 (91.50)	95 (8.15)	4 (0.34)	2227 (95.6)	103 (4.4)		
		SLE (n = 906)	811 (89.51)	93 (10.26)	2 (0.22)	1715 (94.6)	97 (5.4)	0.1649	1.22 [0.92–1.63]

* All P-values have been calculated for the allelic model. ** Pc = 0.248 Benjamini & Hochberg (1995). ***Pc = 9.9E-03 Benjamini & Hochberg (1995) step-up FDR control. ****Odds ratio for the minor allele.

**Figure 1:**
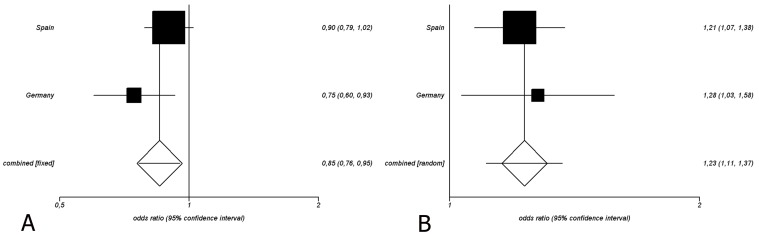
Graphical representation of the meta-analysis (A) Forest plot for the meta-analysis of the *UBASH3a* rs2277798 polymorphism in SLE in two Caucasian cohorts. (**B**) Forest plot for the meta-analysis of the *UBASH3a* rs9976767 polymorphism in SLE in two Caucasian cohorts.

Based on these observations, we evaluated the frequency of the rs9976767 and rs2277798 in a replication cohort from Germany (Table 2). Genotypic and allelic frequencies of both polymorphisms were in HWE. The frequency of the minor allele of both SNPs: rs9976767 and rs2277798 were statistically significant different between SLE patients and healthy controls: rs9976767 (P_c_ =  0.02, OR = 1.28 95%CI = 1.04–1.57) and rs227798 (P_c_ = 0.01, OR = 0.75, 95%CI = 0.6–0.92). Lastly, we combine both the Spanish and German cohorts through a meta-analysis in order to increase the statistical power and to determine the combine OR (Table 3 and Figure 1). This analysis showed evidence of association of the minor allele of rs9976767 with higher SLE risk (P_c_ = 4.7E-03, OR = 1.23 95%CI = 1.11–1.37) and the rs2277798 with lower risk to SLE (P_c_ = 2.4–04, OR = 0.85, 95%CI = 0.76–0.95).

**Table 2:** Genotype and minor allele frequencies of *UBASH3a* SNPs located in Caucasian SLE patients and healthy controls from Germany.

			Genotype, N (%)			Alleles, N(%)		Allele test		
SNP	½	Subgroup (N)	1/1	1/2	2/2	1	2	*P*-value*	*P*_FR_**	OR [CI 95%]***
rs2277798	G/A	Controls (n = 379)	184 (48.55)	132 (34.83)	63 (16.62)	448 (59.1)	310 (40.9)			
		SLE(n = 360)	149 (41.39)	163 (45.28)	48 (13.33)	475 (66)	245 (34)	0.0064	0,0128	0.75 [0.60–0.92]
rs9976767	A/G	Controls (n = 379)	186 (49.08)	136 (35.88)	57 (15.04)	458 (60.4)	300 (39.4)			
		SSc (n = 360)	180 (50.00)	106 (29.44)	74 (20.56)	392 (54.4)	328 (45.6)	0.0201	0,0201	1.28 [1.04–1.57]

* All P-values have been calculated for the allelic model. ** Benjamini & Hochberg (1995) step-up FDR control. ***Odds ratio for the minor allele.

**Table 3:** Meta-analysis of two *UBASH3a* genetic variants within Spanish and German SLE populations.

			Genotype, N (%)			Alleles, N(%)		Allele test		
**SNP**	**½**	**Subgroup (N)**	**1/1**	**1/2**	**2/2**	**1**	**2**	***P*** **-value** *	***P*** _FDR_**	OR [CI 95%]***
rs2277798	G/A	Controls (n = 1544)	609 (39.44)	713 (46.18)	222 (14.38)	1931 (62.5)	1157 (37.5)			
		SLE (n = 1266)	565 (44.63)	543 (42.89)	158 (12.48)	1673 (66.1)	859 (33.9)	0.0047	4.7E-03	0.85 [0.76–0.95]
rs9976767	A/G	Controls (n = 1544)	499 (32.32)	744 (48.19)	301 (19.49)	1742 (56.4)	13446 (43.6)			
		SLE (n = 1266)	336 (26.54)	631 (49.84)	299 (23.62)	1303 (51.5)	1229 (48.5)	1.2E-04	2,4E-04	1.23 [1.11–1.37]

* All P-values have been calculated for the allelic model. **Benjamini & Hochberg (1995) step-up FDR control. ***Odds ratio for the minor allele.

Finally, we prompted out to evaluate whether one of both polymorphisms is responsible for the associations detected using a logistic regression analysis. Pair-wise conditional analysis showed that the association of the rs2277798 SNP was explained by the rs9976767effect, because only the coefficient for the test of rs9976767 remained significant (model conditioned by rs2277798P = 0.76; model conditioned by rs9976767 P = 9E-03, Table 4).

**Table 4:** Conditional logistic regression analysis for two *UBASH3a* SNPs located in SLE considering the two European populations as covariate.

Group of analysis	SNP	MAF Cases	MAF Controls	p Value: add to rs9976767	rs9976767 p value: add to SNP	r2 with rs9976767	
						**Spain**	**Germany**
**SLE**							
	rs2277798	0.34	0.38	0.758	0.0087	0.45	0.41

## Discussion

*UBASH3a* is implicated in the regulation of tyrosine phosphorylation levels within T cells and is involved in facilitates the apoptosis induced in these cells. *UBASH3a* binds to the apoptosis-inducing protein AIF, which has previously been shown to function as a key factor of caspase-independent apoptosis [9]. It has also been reported that SLE T cells, compared with control T cells, undergo an increased rate of apoptosis, which contribute to SLE pathogenesis [4]. Changes in the *UBASH3a* structure or expression levels can affect the binding with AIF leading to an alteration in the apoptosis level.

Herein, we described for the first time the influence of five *UBASH3a* genetic variants in SLE susceptibility. Interestingly, the rs9976767 polymorphism is located in the intronic region between the exons 5 and 6 while the other four studied SNPs (rs2277798, rs2277800, rs13048049 and rs17114930) are non-synonymous changes located in three different exons. The intronic regions flanking constitutive exons contain potential splicing regulatory sequences. Moreover, a study restricted to analysis of the canonical splice signals reported that 15% of point mutations disrupted splicing, a likely gross underestimate of the impact of splicing on human disease [10]. This suggests that the rs9976767 polymorphism could be affecting the expression of different *UBASH3a* isoforms consequently affecting the binding to AIF. Concerning to this we checked if there is any relation between the rs9976767 and expression of *UBASH3a* gene using expression quantitative trait loci (eQTL) databases. Interesting, there is a significant statistical correlation between the increase of *UBASH3a* expression in lymphoblastoid cell lines and the homozygotes for the minor allele of rs9976767 (rho = 0.483, P = 1.3E-05; Figure S2A) in one of the two groups of twins studied (this observation was done using Genevar 3.2.0 software) [11], [12]. Furthermore the eQTL studies in asthma showed that the SNPs (rs9784215, rs3746923, rs2277797) with highest LOD score (LOD>4.5, P<1E-05) in the *UBAHS3a* locus are in moderate to high LD with rs9976767 (Figure S2B and C; this observation was done using mRNA by SNP Browser 1.0.1 http://www.sph.umich.edu/csg/liang/asthma/) [13], [14]. This evidence suggested that rs9976767 could have a functional role in the regulation of the expression of *UBASH3a*. However, and according with HapMap project (http://hapmap.ncbi.nlm.nih.gov/), this SNP tags other six variants in this region (rs7278547, rs11702374, rs9976479, rs3746924, rs3761378, rs7283281; r^2^>0.95) and considering the present study and the previous GWAS [15], [16] we have studied approximately 15% of the genetic variation of *UBASH3a* locus. In order to cover all the genetic variation of this gene, it is necessary to genotype 181 SNPs (calculated through an aggressive tagging with 2-marker haplotypes in Haploview 4.2 software using CEPH population from HapMap project). All these together suggest that the rs9976767 is a good functional candidate risk factor to SLE, but it could be more than one variant related to SLE.

No previous reports have associated the rs9976767 *UBASH3a* polymorphism with SLE. Nevertheless, it is worth noting that the rs9976767 SNP or its six tags variants were not included in previous genome wide association studies (GWAS) in Caucasian SLE cohorts [15], [16]. Although the statistical power is 96% for our meta-analysis (calculate using a p value = 0.05∶OR = 1.2∶ MAF = 0.4), the results found in our study should be replicated in different Caucasian cohorts and other populations. Furthermore there is a need to determine whether the statistical associations are related with the involvement of *UBASH3a* in the pathogenesis of SLE and other autoimmune diseases. Regarding to this, the *UBASH3a* gene seems to be a common genetic factor in autoimmune diseases because different polymorphism of this locus has been associated with autoimmune diseases like T1D, CD, RA and vitiligo [6], [17]–[21]. Our results showed that the minor allele of the rs9976767 *UBASH3a* polymorphism is a risk factor to SLE, as similarly observed with T1D [6]. Nevertheless, there is no evidence of association between this variant and other autoimmune diseases. This can be linked with the suggestion that common genetic factors in autoimmune diseases could match a regional level but differ in the specific genetic variant associated to each disease, like the associations observed with *IL2*–*IL21* and MHC loci [22]. Based on the concept of quantitative thresholds for immune-cell signalling, the effect of the rs976767 *UBAHS3a* variant could diversely affect the range of values for the stimulus-response selection of the immune cells in different autoimmune pathologies, making it more or less relevant in different diseases [3].

In conclusion, our study showed the first evidence of association of the *UBASH3a* gene with the genetic background of SLE. Together the functional role of the protein encoded by this gene, the reported data in the eQTLs databases and our results point to the *UBASH3a* gene as a new element in the pathogenic mechanism of autoimmune diseases.

## Supporting Information

Figure S1 **Pattern of linkage disequilibrium of the five studied SNPs and their location in the UBAHS3a gene.** The values correspond to r2 calculated for the Spanish cohort. The rs2277798 polymorphism [G/A] is located in exon 1 of *UBASH3a* gene. It's a no-synonymous change in the position 18 of the protein (S[Ser]/G[Gly]). The rs2277800 polymorphism [C/T] is also located in exon 1 of *UBASH3a* gene and generate a change in the position 28 of the protein (L[Leu]/F[Phe]). In the other hand, the rs9976767 [A/G] is an intronic variant located between the exons 5 and 6 of the *UBASH3a* gene. Both variants rs13048049 [G/A] and rs17114930 [C/G] are no-synonymous changes in exons 7 and 11, respectively. The first one produce a change from arginine (R[Arg]) to glutamine (Q[Gln]) in position 286; while the rs17114930 polymorphism generates a change from aspartic acid (D[Asp]) to glutamic acid (E[Glu]) in position 428 in Caucasian population.
(TIF)

Figure S2 **Results observed using different expression quantitative trait loci (eQTL)** tools to evaluate if there is any relationship between the rs9976767 variant and the *UBASH3a* expression **(A) SNP-gene association plot** for the rs9976767 and the *UBASH3a* gene based on Spearman's rank correlation coefficient (rho) using the Genevar 3.2 software (http://www.sanger.ac.uk/resources/software/genevar/) [1]. The eQTL analysis was performed in lymphoblastoid cell lines from peripheral blood sample (n =  74). The plot corresponds to one of the two twins groups studied [2]. **(B) Linkage disequilibrium (LD) plot** performed in Haploview 4.2 [3]. LD plot between rs9976767 and the rs9784215, rs3746923, rs2277797 SNPs which exhibited the highest LOD score (LOD>4.5, P<1E-05) in the *UBAHS3a* locus showed in **(C) Snapshot of observed eQTLs related with** ***UBASH3a*** **gene** from the mRNA by SNP Browser 1.0.1 software (http://www.sph.umich.edu/csg/liang/asthma/) based on eQTL studies in asthma [4], [5]. The LOD scores and P values for those SNPs are: rs9784215, LOD = 4.909 P = 2E-06; rs3746923, LOD = 4.905 P = 2E-06; rs2277797, LOD = 4.68 P = 3.4E-06. They are signalled as red dots in the LOD plot. 1. Yang TP, Beazley C, Montgomery SB, Dimas AS, Gutierrez-Arcelus M, et al. (2010) Genevar: a database and Java application for the analysis and visualization of SNP-gene associations in eQTL studies. Bioinformatics 26: 2474-2476. 2. Nica AC, Parts L, Glass D, Nisbet J, Barrett A, et al. (2011) The architecture of gene regulatory variation across multiple human tissues: the MuTHER study. PLoS Genet 7: e1002003. 3. Barrett JC, Fry B, Maller J, Daly MJ (2005) Haploview: analysis and visualization of LD and haplotype maps. Bioinformatics 21: 263-265. 4. Dixon AL, Liang L, Moffatt MF, Chen W, Heath S, et al. (2007) A genome-wide association study of global gene expression. Nat Genet 39: 1202-1207. 5. Moffatt MF, Kabesch M, Liang L, Dixon AL, Strachan D, et al. (2007) Genetic variants regulating ORMDL3 expression contribute to the risk of childhood asthma. Nature 448: 470-473.
(TIF)
