# Supplementary figures and images for: Evidence of New Risk Genetic Factor to Systemic Lupus Erythematosus: The UBASH3A Gene
Source: PLoS One. 2013 Apr 2;8(4):e60646. doi: 10.1371/journal.pone.0060646 (PMC3614928; doi:10.1371/journal.pone.0060646)

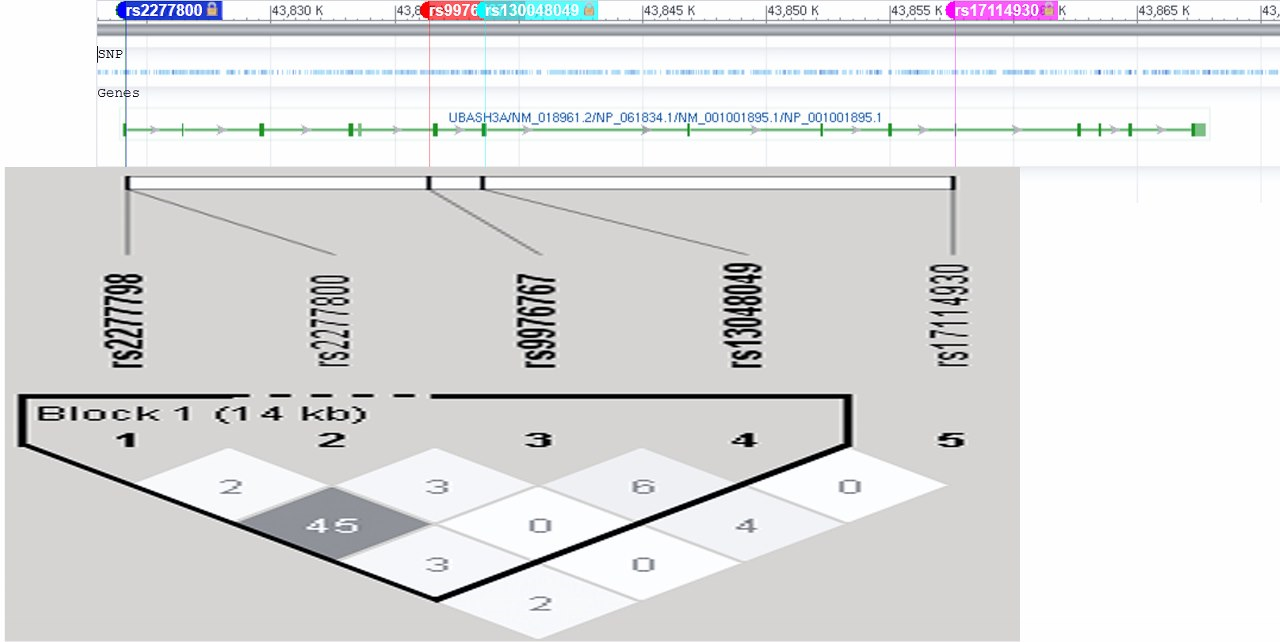

Supplement: Figure S1 — Pattern of linkage disequilibrium of the five studied SNPs and their location in the UBAHS3a gene. The values correspond to r2 calculated for the Spanish cohort. The rs2277798 polymorphism [G/A] is located in exon 1 of UBASH3a gene. It's a no-synonymous change in the position 18 of the protein (S[Ser]/G[Gly]). The rs2277800 polymorphism [C/T] is also located in exon 1 of UBASH3a gene and generate a change in the position 28 of the protein (L[Leu]/F[Phe]). In the other hand, the rs9976767 [A/G] is an intronic variant located between the exons 5 and 6 of the UBASH3a gene. Both variants rs13048049 [G/A] and rs17114930 [C/G] are no-synonymous changes in exons 7 and 11, respectively. The first one produce a change from arginine (R[Arg]) to glutamine (Q[Gln]) in position 286; while the rs17114930 polymorphism generates a change from aspartic acid (D[Asp]) to glutamic acid (E[Glu]) in position 428 in Caucasian population. (TIF) [file pone.0060646.s001.tif]

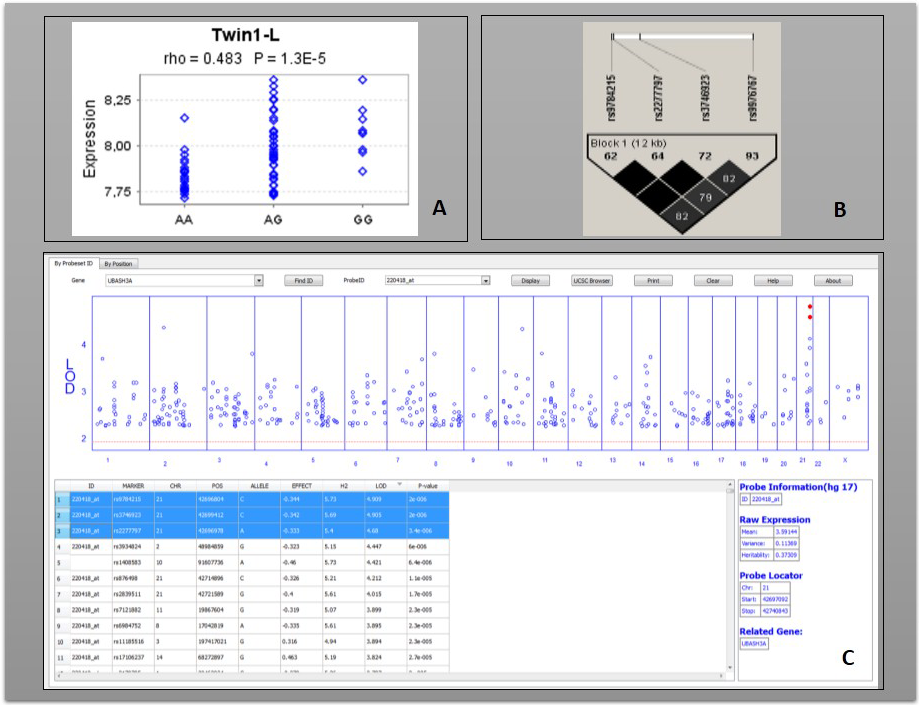

Supplement: Figure S2 — Results observed using different expression quantitative trait loci (eQTL) tools to evaluate if there is any relationship between the rs9976767 variant and the UBASH3a expression (A) SNP-gene association plot for the rs9976767 and the UBASH3a gene based on Spearman's rank correlation coefficient (rho) using the Genevar 3.2 software (http://www.sanger.ac.uk/resources/software/genevar/) [1]. The eQTL analysis was performed in lymphoblastoid cell lines from peripheral blood sample (n = 74). The plot corresponds to one of the two twins groups studied [2]. (B) Linkage disequilibrium (LD) plot performed in Haploview 4.2 [3]. LD plot between rs9976767 and the rs9784215, rs3746923, rs2277797 SNPs which exhibited the highest LOD score (LOD>4.5, P<1E-05) in the UBAHS3a locus showed in (C) Snapshot of observed eQTLs related with UBASH3a gene from the mRNA by SNP Browser 1.0.1 software (http://www.sph.umich.edu/csg/liang/asthma/) based on eQTL studies in asthma [4], [5]. The LOD scores and P values for those SNPs are: rs9784215, LOD = 4.909 P = 2E-06; rs3746923, LOD = 4.905 P = 2E-06; rs2277797, LOD = 4.68 P = 3.4E-06. They are signalled as red dots in the LOD plot. 1. Yang TP, Beazley C, Montgomery SB, Dimas AS, Gutierrez-Arcelus M, et al. (2010) Genevar: a database and Java application for the analysis and visualization of SNP-gene associations in eQTL studies. Bioinformatics 26: 2474-2476. 2. Nica AC, Parts L, Glass D, Nisbet J, Barrett A, et al. (2011) The architecture of gene regulatory variation across multiple human tissues: the MuTHER study. PLoS Genet 7: e1002003. 3. Barrett JC, Fry B, Maller J, Daly MJ (2005) Haploview: analysis and visualization of LD and haplotype maps. Bioinformatics 21: 263-265. 4. Dixon AL, Liang L, Moffatt MF, Chen W, Heath S, et al. (2007) A genome-wide association study of global gene expression. Nat Genet 39: 1202-1207. 5. Moffatt MF, Kabesch M, Liang L, Dixon AL, Strachan D, et al. (2007) Genetic variants regulating ORMDL3 expression contrib [file pone.0060646.s002.tif]
